# Supplementary material for: ANGPTL4 exacerbates pancreatitis by augmenting acinar cell injury through upregulation of C5a
Source: EMBO Mol Med. 2020 Jul 7;12(8):e11222. doi: 10.15252/emmm.201911222 (PMC7411571; doi:10.15252/emmm.201911222)
Supplement: Supplementary file 1 — Appendix [file EMMM-12-e11222-s001.pdf]

Appendix

ANGPTL4 exacerbates pancreatitis by augmenting acinar cell injury through upregulation of C5a.

Kyung Hee Jung[1,†], Mi Kwon Son[1,†], Hong Hua Yan[1], Zhenghuan Fang[1], Ju Young Kim[1], Soo Jung Kim[1], Jung Hee Park[1], Ji Eun Lee[1], Young-Chan Yoon[1], Myeong Seong Seo[1], Beom Seok Han[1], Soyeon Ko[1], Young Ju Suh[1], Ju Han Lim[1], Don-Haeng Lee[1], Ziqiang Teo[2], Jonathan Wei Kiat Wee[2], Nguan Soon Tan[2,3,*], Soon–Sun Hong[1,*]

Contents

1. Appendix Table S1- S2

2. Appendix Figures S1-S8

Appendix Table S1. Information of genes and primers used for qRT-PCR

Name	Accession No.	Forward primer sequence (5'-3')	Reverse primer sequence (5'-3')	Amplicon size
TNF-α	NM_013693	AACGGCATGGATCTCAAAGA	GCCTTGTCCCTTGAAGAGAA	90 bp
IL-6	NM_031168	AGAGGATACCACTCCCAACA	CACGATTTCCCAGAGAACAT	82 bp
IL-1β	NM_ 008361	AGAGTTCCCCAACTGGTACA	TCCTGACCACTGTTGTTTCC	78 bp
C5a	NM_010406.3	GGGAAGCTGCTGATGAGAAT	CTTTCACCAGGTTGGCATTG	75 bp
C5aR	NM_001173550	GTGTC CCTGGCCTACATCAA	AGGAGTCGTCCATGGAAACC	80 bp
GAPDH	NM_008084.3	GGCATTGCTCTCAATGACAA	ATGTAGGCCATGAGGTCCAC	95 bp

Appendix Table S2. Exact P-values and statistical test

Figure	Name	Compared groups	P-value	Test
Fig.1A	Amylase	Normal vs Pancreatitis patient	0.007	Mann-Whitney U-test
	Lipase	〃	0.001	Mann-Whitney U-test
	CRP	〃	5.68048E-17	Mann-Whitney U-test
	ANGPTL4	〃	1.08569E-06	Mann-Whitney U-test
Fig.1D	Amylase	Con vs AP, Con vs SAP	9.18948E-21<br>4.21967E-25	One-way ANOVA
	Lipase	〃	7.97889E-18<br>2.17314E-17	One-way ANOVA
	ANGPTL4	〃	2.57677E-18<br>8.2086E-19	One-way ANOVA
Fig.2B	TGF-β	SAP vs ANGPTL4	1.04251E-07	Unpaired T-test
	IL-1β	〃	0.041	Unpaired T-test
	IFN-γ	〃	3.15339E-04	Unpaired T-test
	ANGPTL4	〃	3.22724E-06	Unpaired T-test
Fig.3B	ANGPTL4	ANGPTL4(-/-) AP and SAP<br>vs WT AP and SAP	1.00325E-09<br>1.24463E-14	One-way ANOVA
	Amylase	〃	1.50672E-30<br>4.95166E-26	One-way ANOVA
	MPO	〃	1.4248E-10<br>5.26087E-15	One-way ANOVA
	Lipase	〃	8.70038E-17<br>2.60837E-20	One-way ANOVA
Fig.3C	TGF-β	〃	9.88989E-09<br>3.28829E-12	One-way ANOVA
	IFN-γ	〃	9.3311E-08<br>2.44151E-10	One-way ANOVA
	IL-1β	〃	9.29602E-10<br>2.19704E-11	One-way ANOVA
Fig.4A	Amylase	Macrophage$^{+/+}$ AP<br>vs Macrophage$^{-/-}$ AP	3.04822E-12	One-way ANOVA
		Macrophage$^{+/+}$ SAP<br>vs Macrophage$^{-/-}$ SAP	7.35318E-10	One-way ANOVA
		Macrophage$^{+/+}$ AP+ANGPTL4<br>vs Macrophage$^{-/-}$ AP+ANGPTL4	1.73994E-12	One-way ANOVA
		Macrophage$^{+/+}$ ANGPTL4 (4mg/kg)<br>vs Macrophage$^{-/-}$ ANGPTL4 (4mg/kg)	1.91997E-14	One-way ANOVA

Figure	Name	Compared groups	P-value	Test
Fig.4A	Lipase	Macrophage$^{+/+}$ AP vs Macrophage$^{-/-}$ AP	2.29964E-06	One-way ANOVA
		Macrophage$^{+/+}$ SAP vs Macrophage$^{-/-}$ SAP	2.68295E-13	One-way ANOVA
		Macrophage$^{+/+}$ AP+ANGPTL4 vs Macrophage$^{-/-}$ AP+ANGPTL4	1.00306E-14	One-way ANOVA
		Macrophage$^{+/+}$ ANGPTL4 (4mg/kg) vs Macrophage$^{-/-}$ ANGPTL4 (4mg/kg)	3.55562E-14	One-way ANOVA
Fig.4B	ANGPTL4	Macrophage$^{+/+}$ AP vs Macrophage$^{-/-}$ AP	2.44016E-06	One-way ANOVA
		Macrophage$^{+/+}$ SAP vs Macrophage$^{-/-}$ SAP	4.07255E-11	One-way ANOVA
		Macrophage$^{+/+}$ANGPTL4 vs Macrophage$^{-/-}$ ANGPTL4	1.39395E-13	One-way ANOVA
Fig.4C	Macrophage positive cells	ANGPTL4 WT AP vs ANGPTL4$^{-/-}$ AP	3.36932E-06	One-way ANOVA
		ANGPTL4 WT SAP vs ANGPTL4$^{-/-}$ SAP	8.29676E-11	One-way ANOVA
	TNF-α	Con vs LPS	0.001058	One-way ANOVA
		Con vs ANGPTL4	6.34417E-05	One-way ANOVA
Fig.5A	IL-6	Con vs LPS	3.344109E-05	One-way ANOVA
		Con vs ANGPTL4	2.50579E-05	One-way ANOVA
	IL-1β	Con vs LPS	0.000151	One-way ANOVA
		Con vs ANGPTL4	0.000385	One-way ANOVA
	TGF-β	Con vs LPS	2.40382E-08	One-way ANOVA
		Con vs ANGPTL4	9.43636E-08	One-way ANOVA
	NO	Con vs LPS	2.16979E-06	One-way ANOVA
		Con vs ANGPTL4	3.90836E-08	One-way ANOVA
Fig.5B	Migrated cells	Con vs CCK	0.005	One-way ANOVA
		Con vs LPS	2.02756E-06	One-way ANOVA
		Con vs ANGPTL4	3.59717E-06	One-way ANOVA
Fig.7A	Human C5a	Normal vs Pancreatitis patient	4.21089E-11	Mann-Whitney U-test
Fig.7B	C5a	ANGPTL4 WT AP vs ANGPTL4$^{-/-}$ AP	0.002	One-way ANOVA
		ANGPTL4 WT SAP vs ANGPTL4$^{-/-}$ SAP	6.0688E-07	One-way ANOVA

Figure	Name	Compared groups	P-value	Test
Fig.7B	C5a	Macrophage$^{+/+}$ AP vs Macrophage$^{-/-}$ AP	0.00027	One-way ANOVA
		Macrophage$^{+/+}$ SAP vs Macrophage$^{-/-}$ SAP	4.53221E-06	One-way ANOVA
Fig.7C	ANGPTL4	LPS vs LPS + nANGPTL4 Ab	1.3594E-07	One-way ANOVA
		LPS vs LPS + nc5a Ab	7.33199E-06	One-way ANOVA
	C5a	LPS vs LPS + nANGPTL4 Ab	0.000119	One-way ANOVA
		LPS vs LPS + nc5a Ab	5.3672E-06	One-way ANOVA
	TNF-α	LPS vs LPS + nANGPTL4 Ab	5.38054E-07	One-way ANOVA
		LPS vs LPS + nc5a Ab	1.27613E-06	One-way ANOVA
	IL-6	LPS vs LPS + nANGPTL4 Ab	1.70199E-05	One-way ANOVA
		LPS vs LPS + nc5a Ab	0.000165	One-way ANOVA
Fig.7D	ANGPTL4	AP vs AP + nANGPTL4 Ab	6.79258E-06	One-way ANOVA
		SAP vs SAP + nANGPTL4 Ab	4.0549E-08	One-way ANOVA
	C5a	AP vs AP + nANGPTL4 Ab	1.89725E-09	One-way ANOVA
		SAP vs SAP + nANGPTL4 Ab	2.79241E-06	One-way ANOVA
	TNF-α	AP vs AP + nANGPTL4 Ab	2.02773E-07	One-way ANOVA
		SAP vs SAP + nANGPTL4 Ab	9.66435E-09	One-way ANOVA
	Amylase	AP vs AP + nANGPTL4 Ab	3.76754E-09	One-way ANOVA
		SAP vs SAP + nANGPTL4 Ab	2.35263-07	One-way ANOVA

Appendix Fig S1

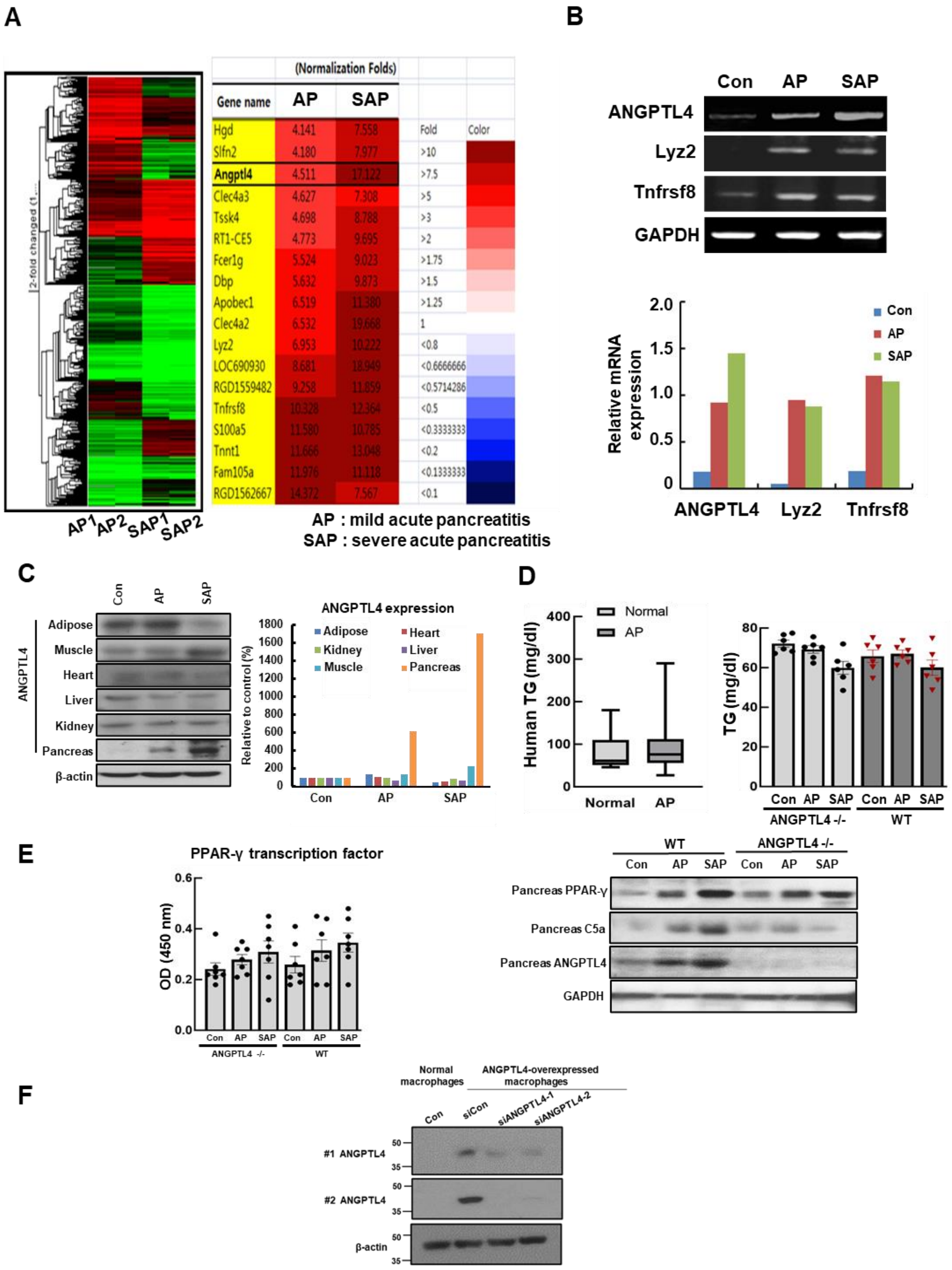

Appendix Fig S2

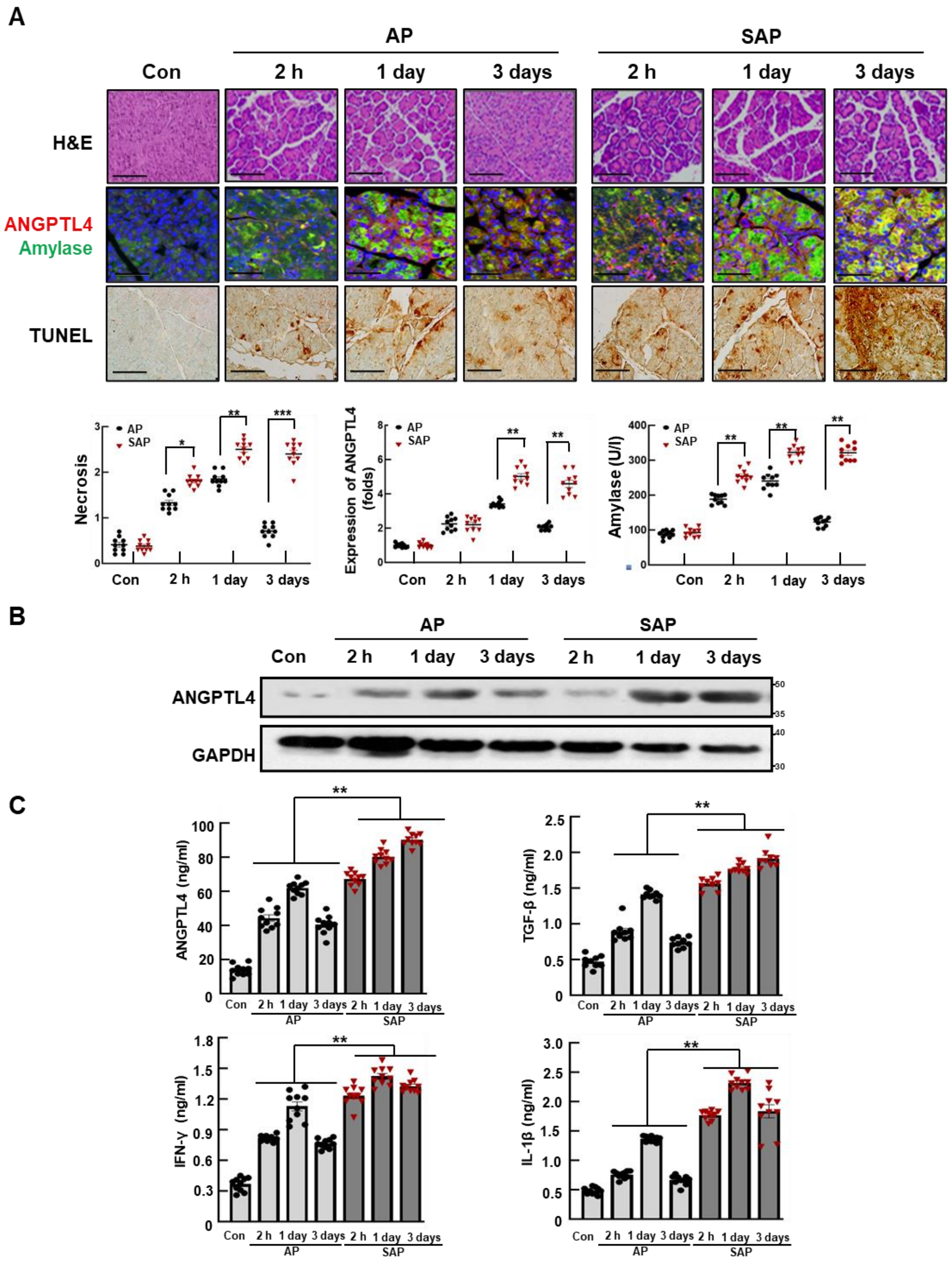

Appendix Fig S3

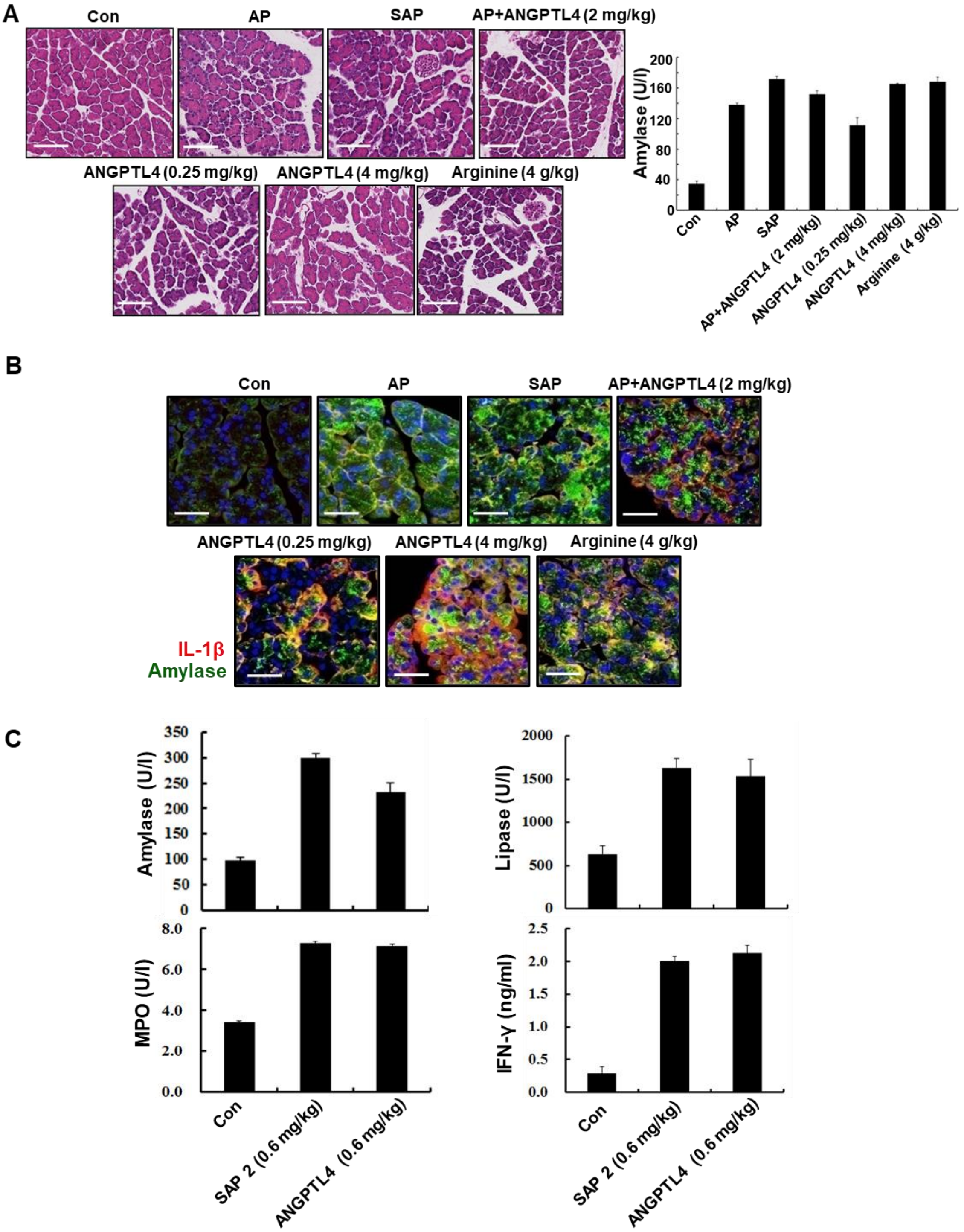

Appendix Fig S4

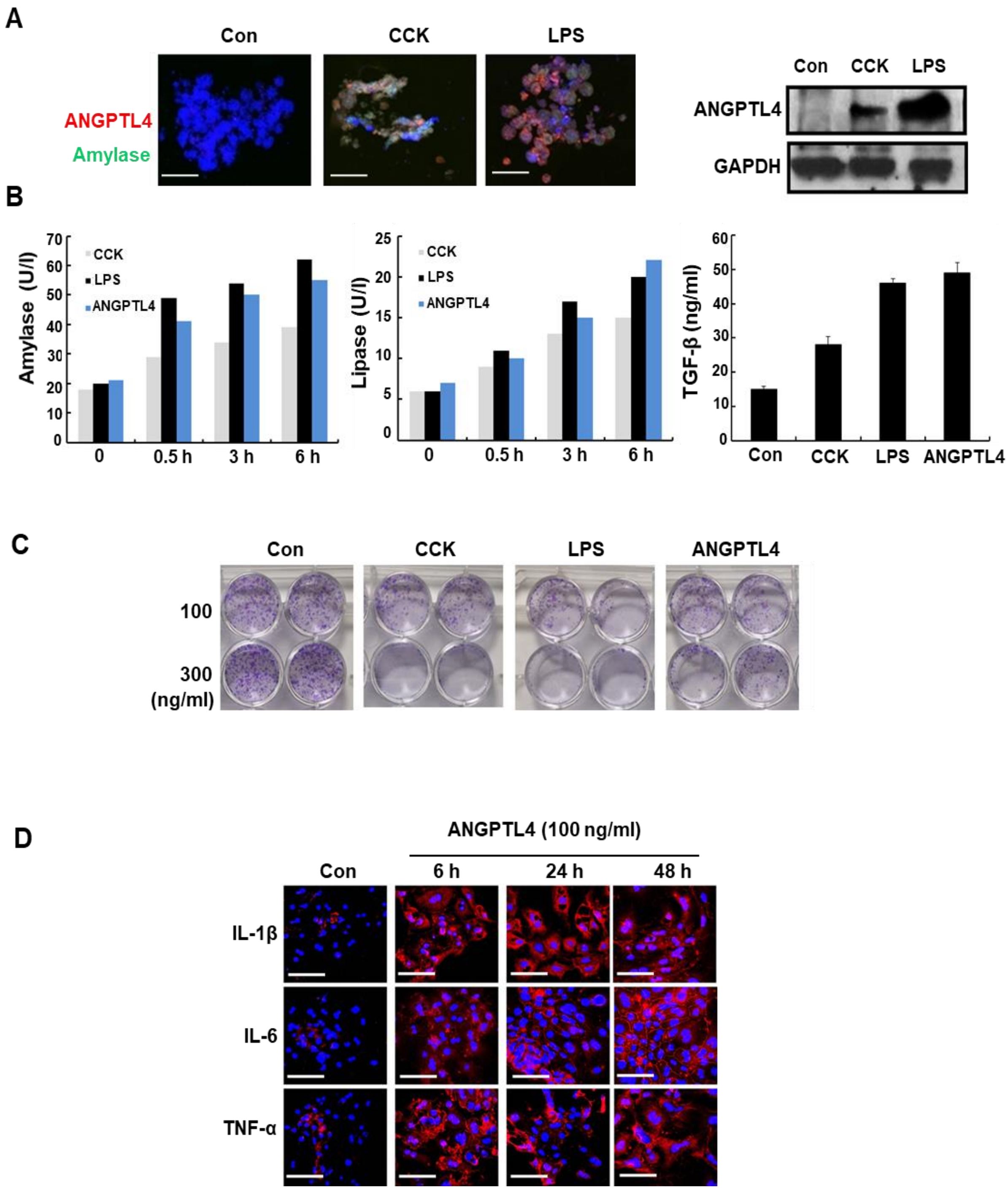

Appendix Fig S5

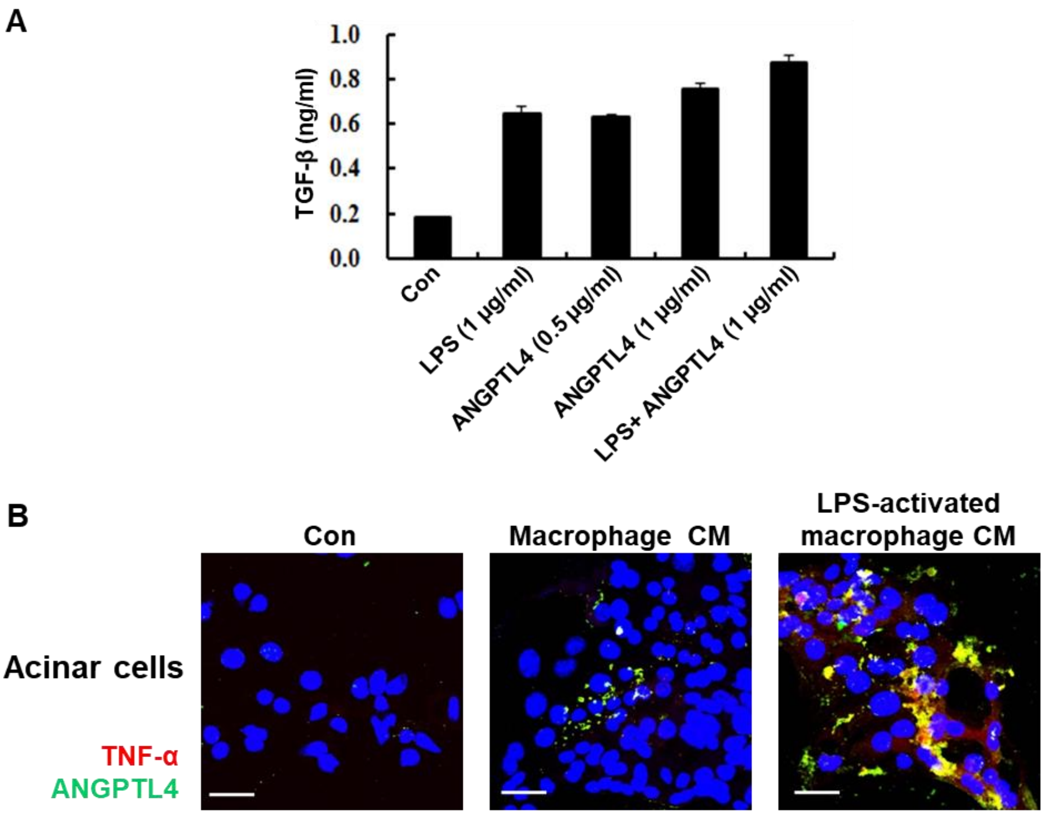

Appendix Fig S6

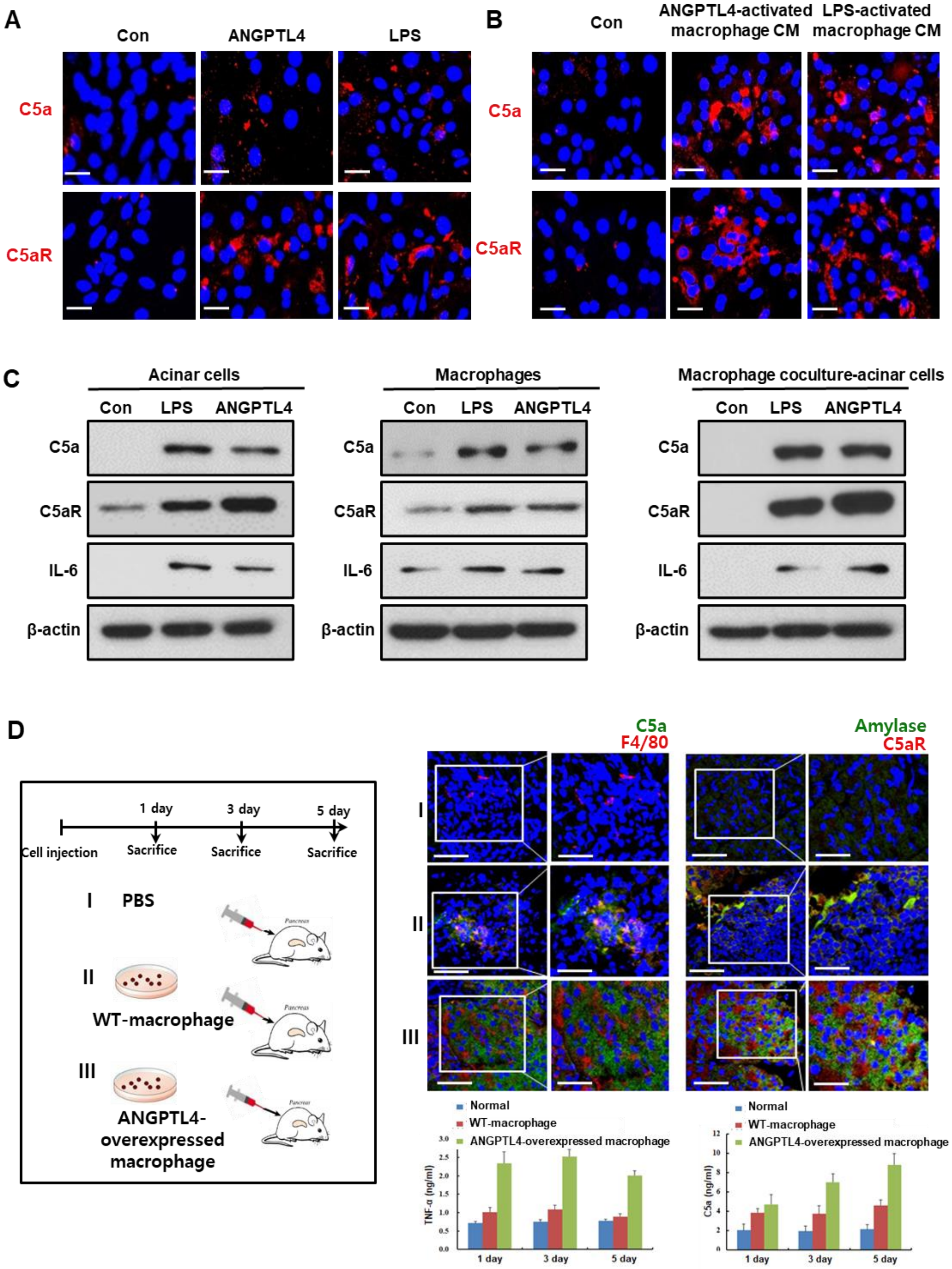

Appendix Fig S7

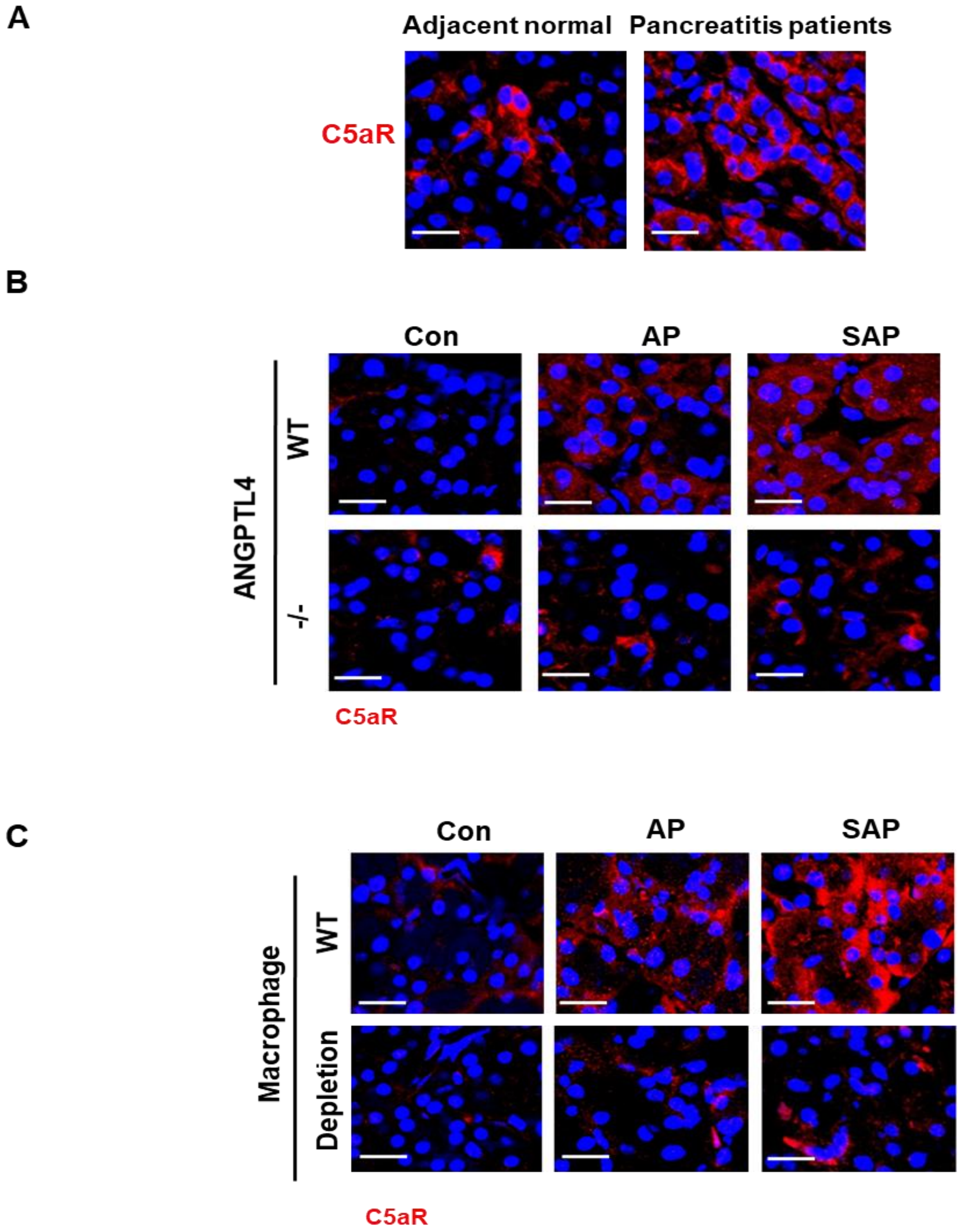

Appendix Fig S8

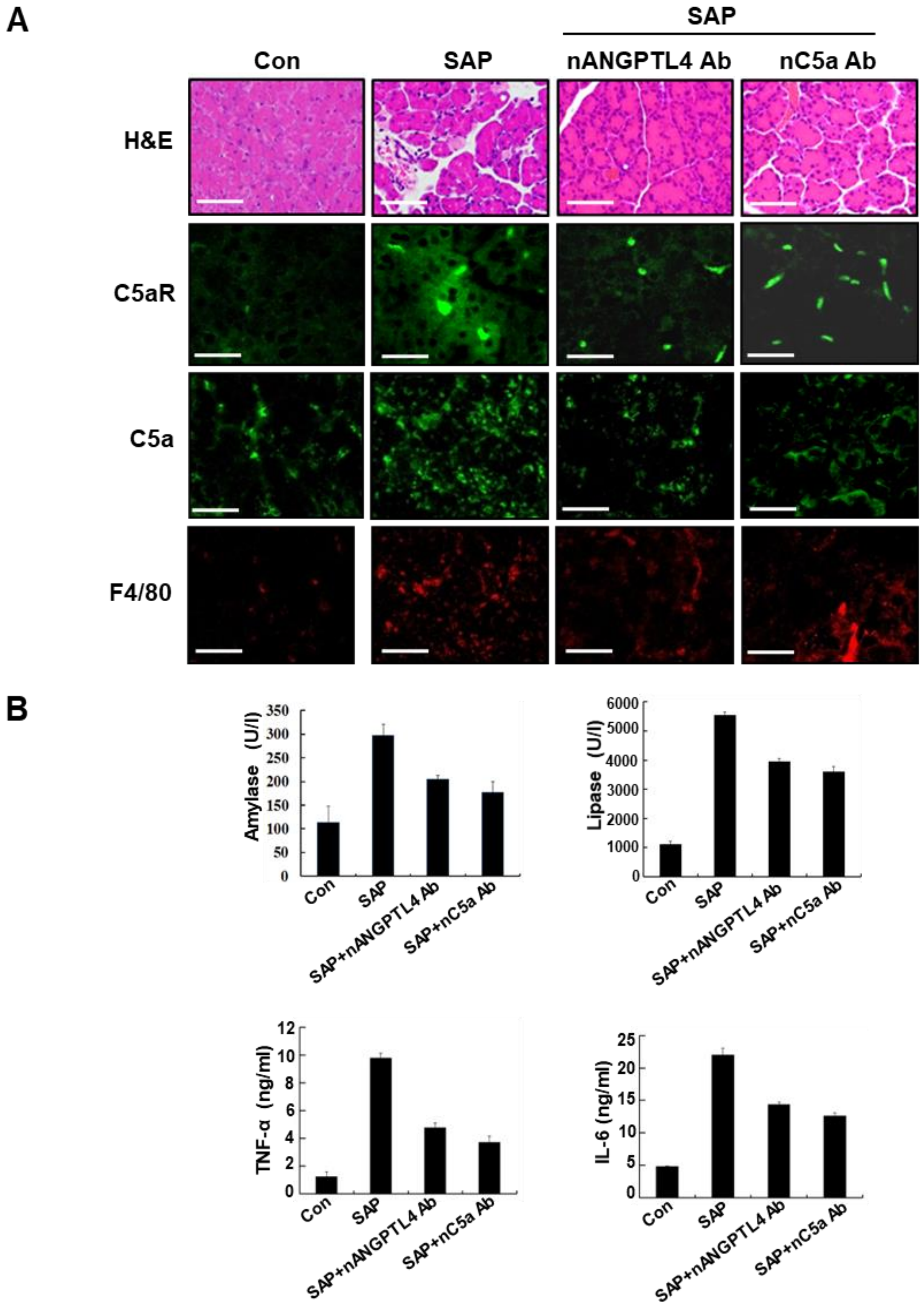

Appendix Figure legends

Appendix Figure S1. Gene profiling analysis and related signaling in pancreatitis animal model.

A Hierarchical cluster analysis of microarray gene expression in pancreas isolated from mild (AP) and severe acute pancreatitis (SAP) animal models. Eighteen genes (>4-fold) were selected to generate a heat map.

B The mRNA expression and quantification of ANGPTL4, Lyz2, and Tnfrsf8.

C ANGPTL4 expression in multi-organs 24 h after AP and SAP induction (n = 3-5).

D The TG level were evaluated by ELISA in human pancreatitis patient (n=80) and ANGPTL4 -/- and WT mice model (n=6).

E PPAR-γ level and its transcription level in pancreas nucleic protein of ANGPTL4 -/- and WT mice using PPAR-γ transcription factor assay kit. Expression of PPAR-γ, C5a, and ANGPTL4 in pancreas were determined by western blot analysis.

F To identify the specificity of the ANGPTL4 antibody, the expression of ANGPTL4 after the knockdown using two siANGPTL4 was checked in ANGPTL4-overexpressed macrophages.

Data information: Each value represents the mean ± SEM.

Appendix Figure S2. Pattern of ANGPTL4 expression in time-dependent pancreatitis model.

A Pancreatic tissues were harvested at 2 h, 1 day, and 3 days after AP and SAP induction. H&E, immunofluorescence staining for ANGPTL4 (red) and amylase (green), and TUNEL staining were performed in the pancreatic tissues. Levels of amylase and ANGPTL4 in the serum and necrosis in the pancreas tissues were evaluated by ELISA and pathological analysis (n = 10, each group). Scale bar represents 50 μm.

B Protein expression of ANGPTL4 in pancreatic tissues by Western blotting.

C Levels of inflammatory cytokines including TGF-β, IL-1β, IFN-γ, and ANGPTL4 in the serum (n = 10, each group).

Data information: Each value represents the mean ± SEM. (*P < 0.05 and ** P < 0.01). Values of P were calculated using one-way ANOVA with Tukey's post-hoc analysis.

Appendix Figure S3. Pancreatitis induced by ANGPTL4.

A AP was induced by 5 injections (IP) of cerulein at a dosage of 50 μg/kg. SAP was induced by 5 hourly injections of cerulein at a dosage of 50 μg/kg and one injection of lipopolysaccharide (LPS, IP) at a dosage of 20 mg/kg. The AP+ANGPTL4 (2 mg/kg) group was induced by AP and one injection of ANGPTL4 (IP) at a dosage of 2 mg/kg. The ANGPTL4 (0.25 mg/kg) group was induced by 5 injections (IP) of ANGPTL4 at a dosage of 50 μg/kg, which is same dose with cerulein for the induction of AP. The ANGPTL4 (4 mg/kg) group was induced by 2 injections (IP) of ANGPTL4 at dosage of 2 mg/kg, and arginine group was induced by two injections of arginine (IP) at a dosage of 2 g/kg body weight. All the mice were sacrificed 6 h after pancreatitis induction. The levels of amylase in the serum were evaluated by ELISA. Scale bars, 100 μm.

B Immunofluorescence staining for IL-1β (red) and amylase (green) was performed in the pancreatic tissues. Scale bars, 100 μm.

C Levels of amylase, lipase, MPO, and IFN-γ in serum were measured in the SAP 2 (0.6 mg/kg) and ANGPTL4 (0.6 mg/kg) groups.

Data are represented as the mean ± SEM.

Appendix Figure S4. Amylase and lipase activity after CCK, LPS and ANGPTL4 treatment to acinar cells.

A Immunofluorescence staining and protein expression of ANGPTL4 in acinar cells after CCK and LPS treatment for 6 h. Scale bars, 50 μm.

B The levels of amylase and lipase at 0, 0.5, 3, and 6 h after CCK, LPS, and ANGPTL4 (0.1 μM) treatment. The levels of TGF-β were measured in media from pancreas acinar cells after CK, LPS, and ANGPTL4 (0.1 μM) treatment for 24 h.

C Viability assay was performed in mouse primary acinar cells treated with 100 and 300 ng/ml CCK, LPS and ANGPTL4 for 72 h. Cells were stained with crystal violet.

D Expression of inflammatory cytokines in pancreas acinar cells after ANGPTL4 (100 ng/ml)

treatment. Scale bars, 50 μm. Data are represented as the mean ± SEM.

Appendix Figure S5. Effect of ANGPTL4 on macrophages and acinar cells.

A Peritoneal macrophages were freshly isolated and activated with LPS and ANGPTL4, and then TGF-β levels were measured in culture media by ELISA. Data are represented as the mean ± SEM.

B Mouse macrophages were activated by LPS (100 ng/ml) for 24 h. Acinar cells were treated with macrophage-conditioned media (CM) and LPS-activated macrophage CM for 6 h. Immunofluorescence staining for TNF-α (red) and ANGPTL4 (green) was performed in macrophage CM-treated pancreatic acinar cells. Scale bars, 30 μm.

Appendix Figure S6. Expression of C5a and C5aR by ANGPTL4-activated macrophage and its *in vivo* effect on the progression of AP.

A Immunofluorescence staining of C5a and C5aR in pancreatic acinar cells after treatment with ANGPTL4 or LPS (100 ng/ml) for 6 h. Scale bars, 30 μm

B Mouse macrophages were activated by ANGPTL4 or LPS (100 ng/ml) for 24 h. Acinar cells were treated with macrophage conditioned media (CM) and ANGPTL4- and LPS-activated macrophage CM for 6 h. After treatment with CM, the expression of C5a and C5aR was identified in the pancreatic acinar cells. Scale bars, 30 μm.

C C5a, C5aR, and IL-6 expression was identified in mouse macrophages or primary acinar cells after treatment with ANGPTL4 or LPS (100 ng/ml). In the transwell experiment, mouse primary acinar cells were cultured in the lower well and macrophages were incubated in the upper transwell bucket treated with ANGPTL4.

D AP severity by pancreas injection of ANGPTL4-overexpressed macrophages. Macrophages and ANGPTL4-overexpressed macrophages (5 x105 cells/mouse) were injected into pancreas. Mice

were sacrificed on 1 day, 3 day, and 5 day after cell injection. Levels of TNF-α and C5a were measured in serum. Pancreas tissues were obtained on 5 day after cell injection and then were stained with F4/80, C5a, amylase, and C5aR. Scale bars, 30 μm.

Data are represented as the mean ± SEM.

Appendix Figure S7. Expression of C5aR by ANGPTL4.

A Immunofluorescence staining of C5aR in pancreatitis and adjacent normal regions of patient tissues. Scale bars, 30 μm.

B The expression of C5aR in tissues from ANGPTL4 WT and ANGPTL4 -/- mice after AP and SAP induction. Scale bars, 30μm.

C The expression of C5aR in pancreas tissues isolated from WT and depleted macrophages after AP and SAP induction. Scale bars, 30 μm.

Appendix Figure S8. Therapeutic effect of neutralizing ANGPTL4 or C5a antibody *in vivo*.

A Neutralizing ANGPTL4 or C5a antibody (10 mg/kg) was injected twice (IP) into the SAP models. H&E staining and the expression of C5a, C5aR and F4/80 in pancreatic tissues. Scale bars, 50 μm.

B Level of amylase, lipase, TNF-α and IL-6 in the serum. Data are represented as the mean ± SEM.
