# Supplementary figures and images for: ANGPTL4 exacerbates pancreatitis by augmenting acinar cell injury through upregulation of C5a
Source: EMBO Mol Med. 2020 Jul 7;12(8):e11222. doi: 10.15252/emmm.201911222 (PMC7411571; doi:10.15252/emmm.201911222)

ANGPTL4 antibody specificity (supplementary figure 1F)

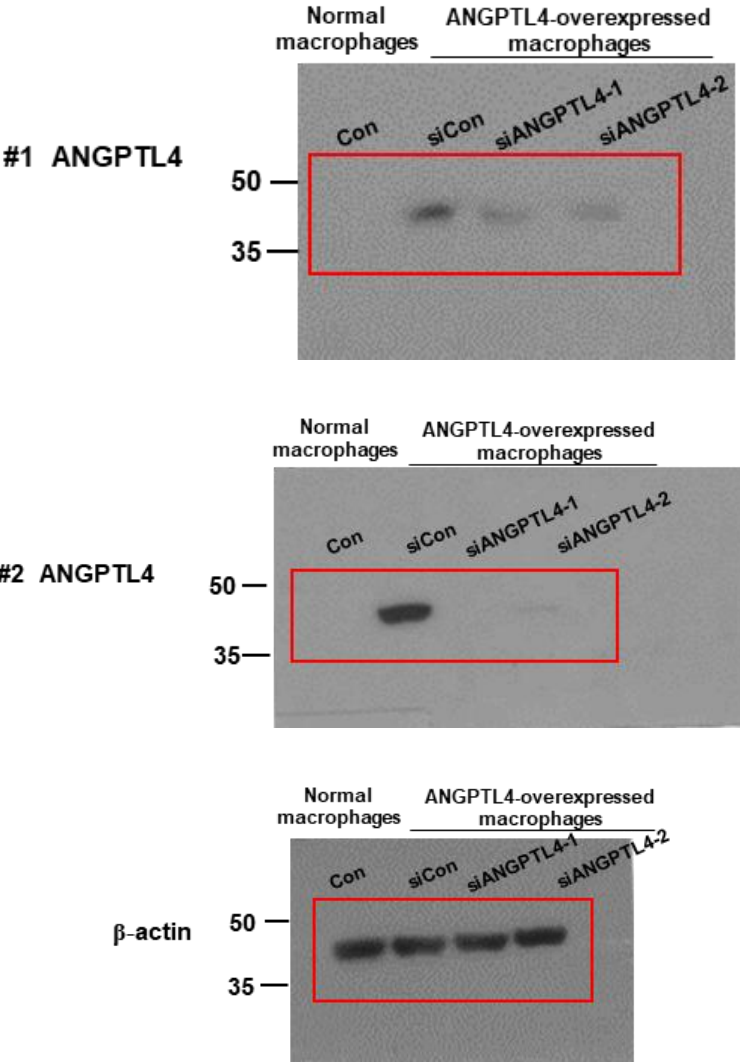

Supplement: Supplementary file 2 — Source Data for Appendix [file EMMM-12-e11222-s006.zip › EMM-2019-11222-V5-Figure_Source_Data-FigS1.pdf]

**Figure 1D**

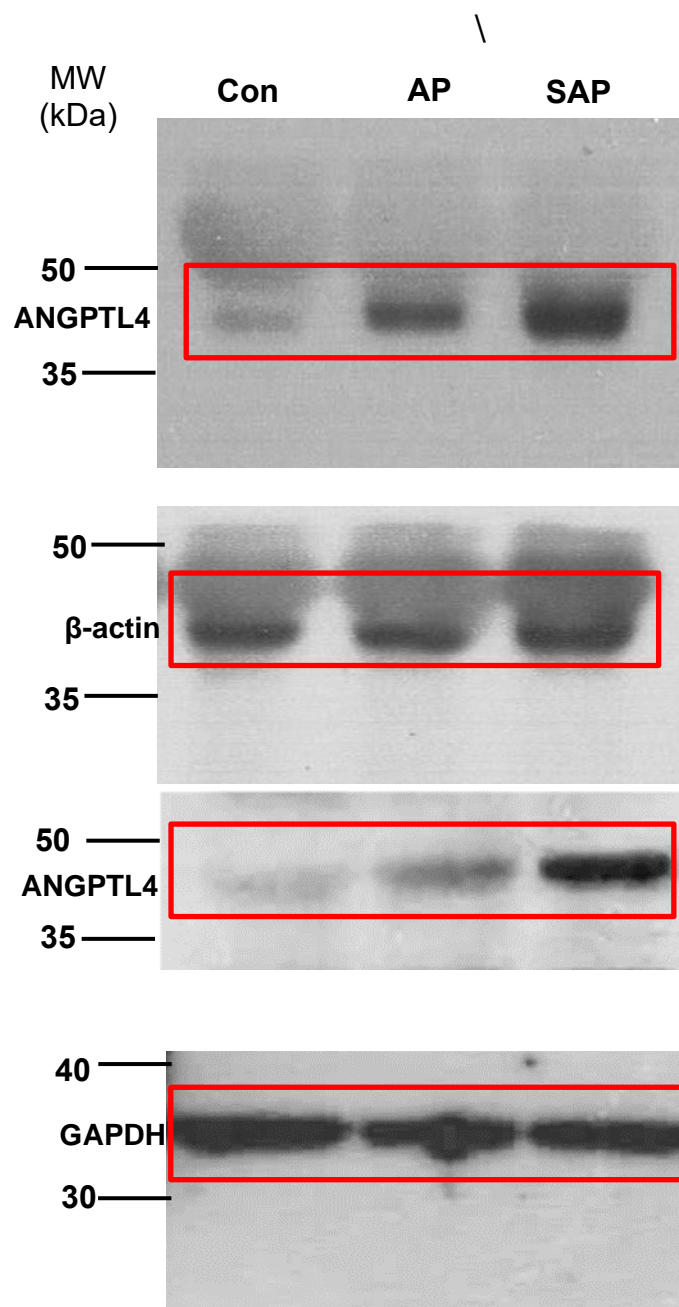

Supplement: Supplementary file 4 — Source Data for Figure 1 [file EMMM-12-e11222-s002.pdf]

**Figure 3A**

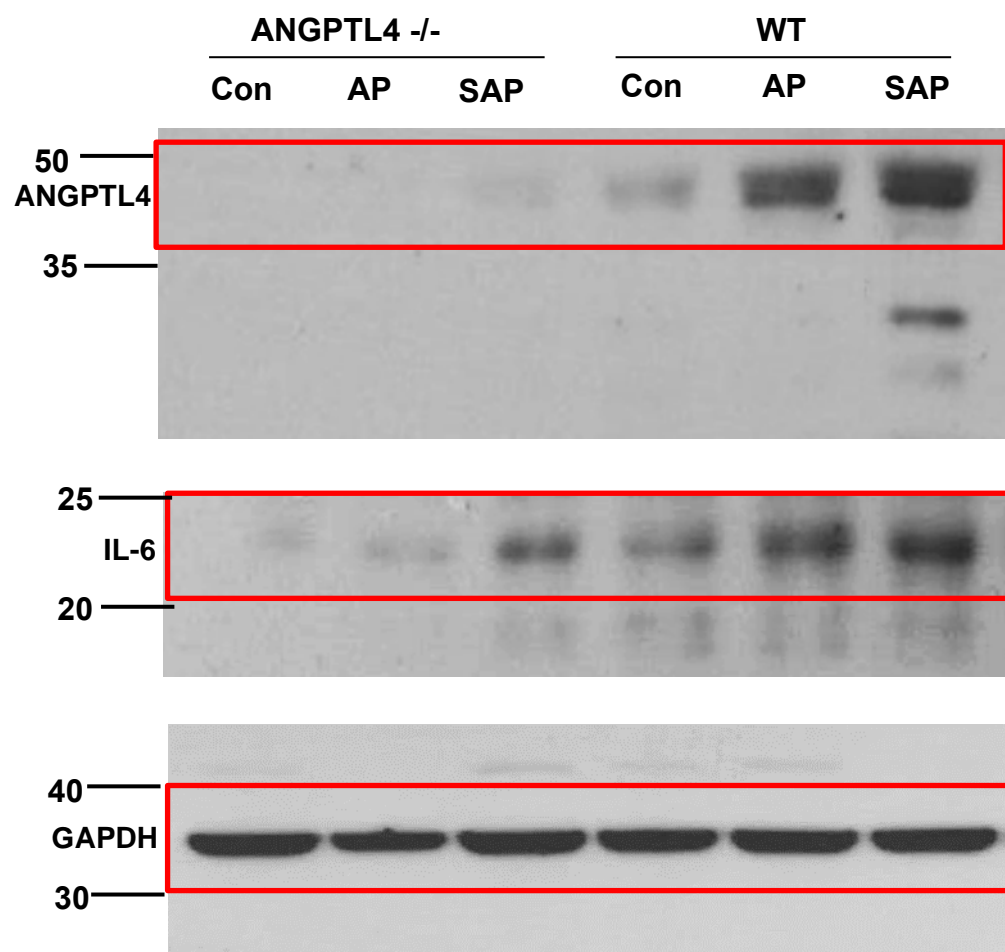

Supplement: Supplementary file 5 — Source Data for Figure 3 [file EMMM-12-e11222-s003.pdf]

**Figure 5A raw data**

**IL-6**

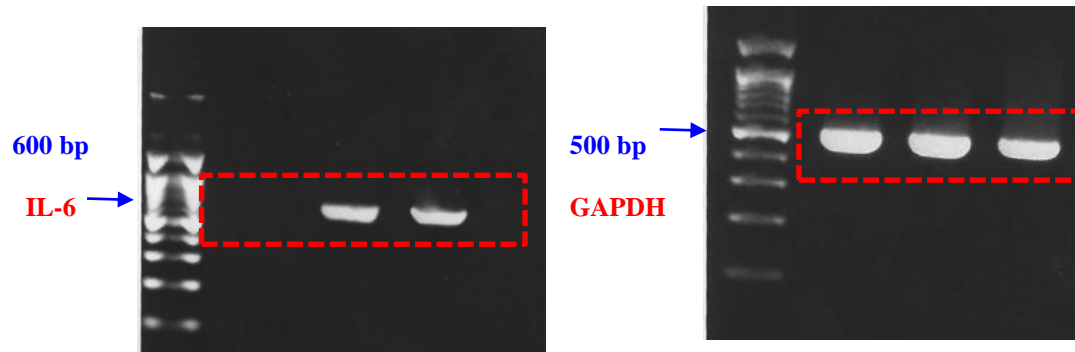

**IL-1 $\beta$**

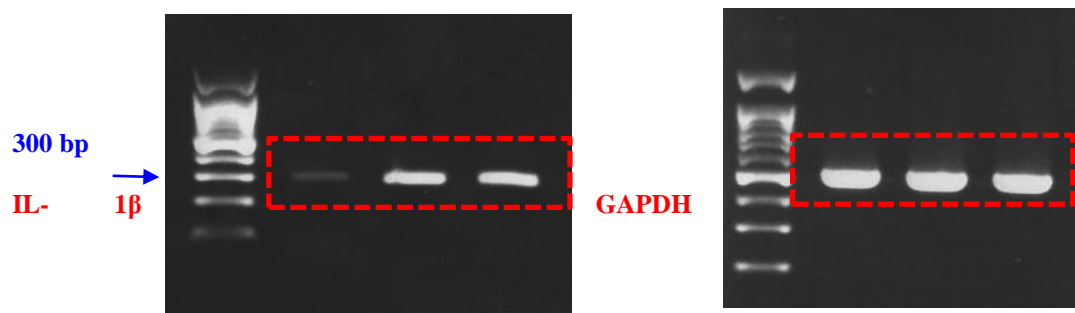

**TNF- $\alpha$**

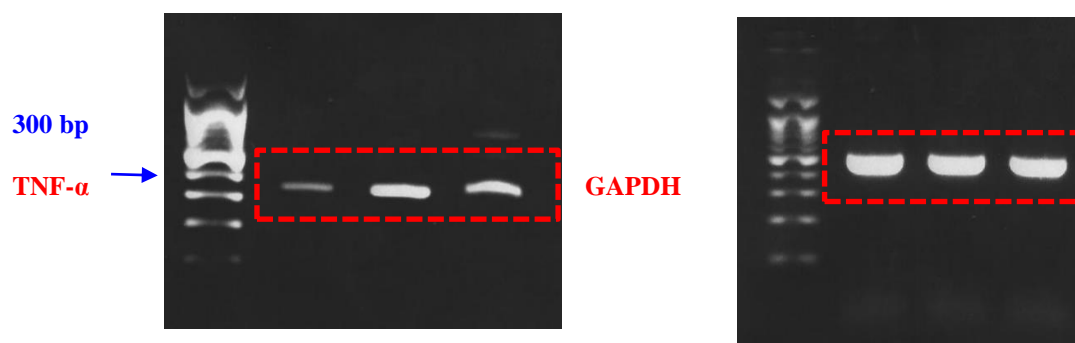

Supplement: Supplementary file 6 — Source Data for Figure 5 [file EMMM-12-e11222-s004.pdf]

**Figure 6B**

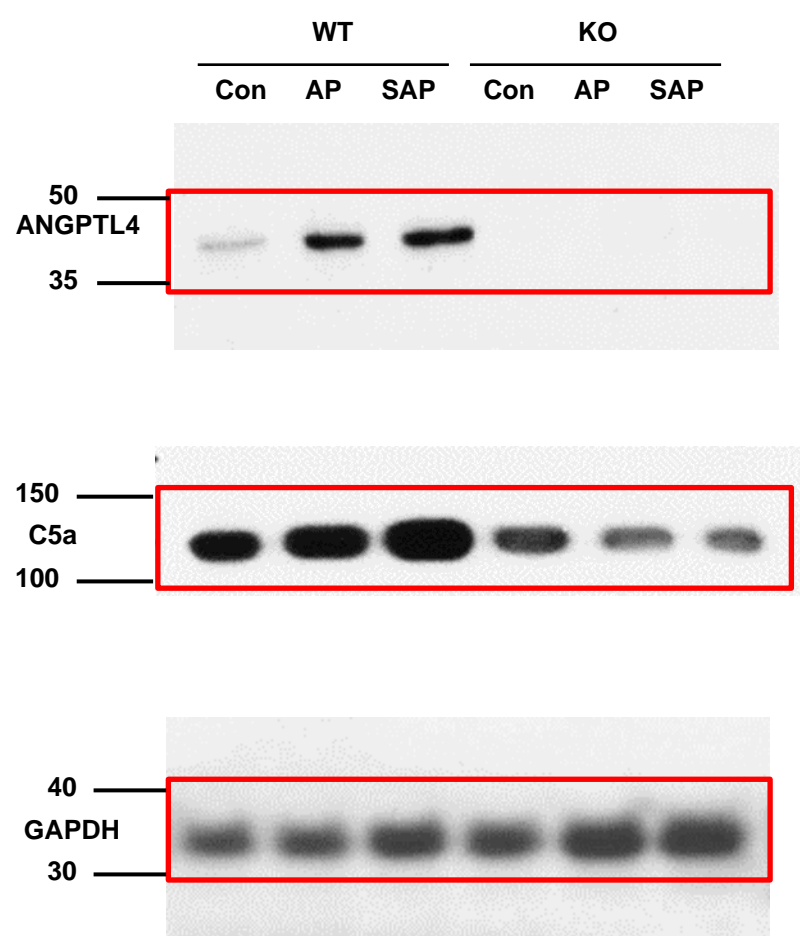

**Figure 6C**

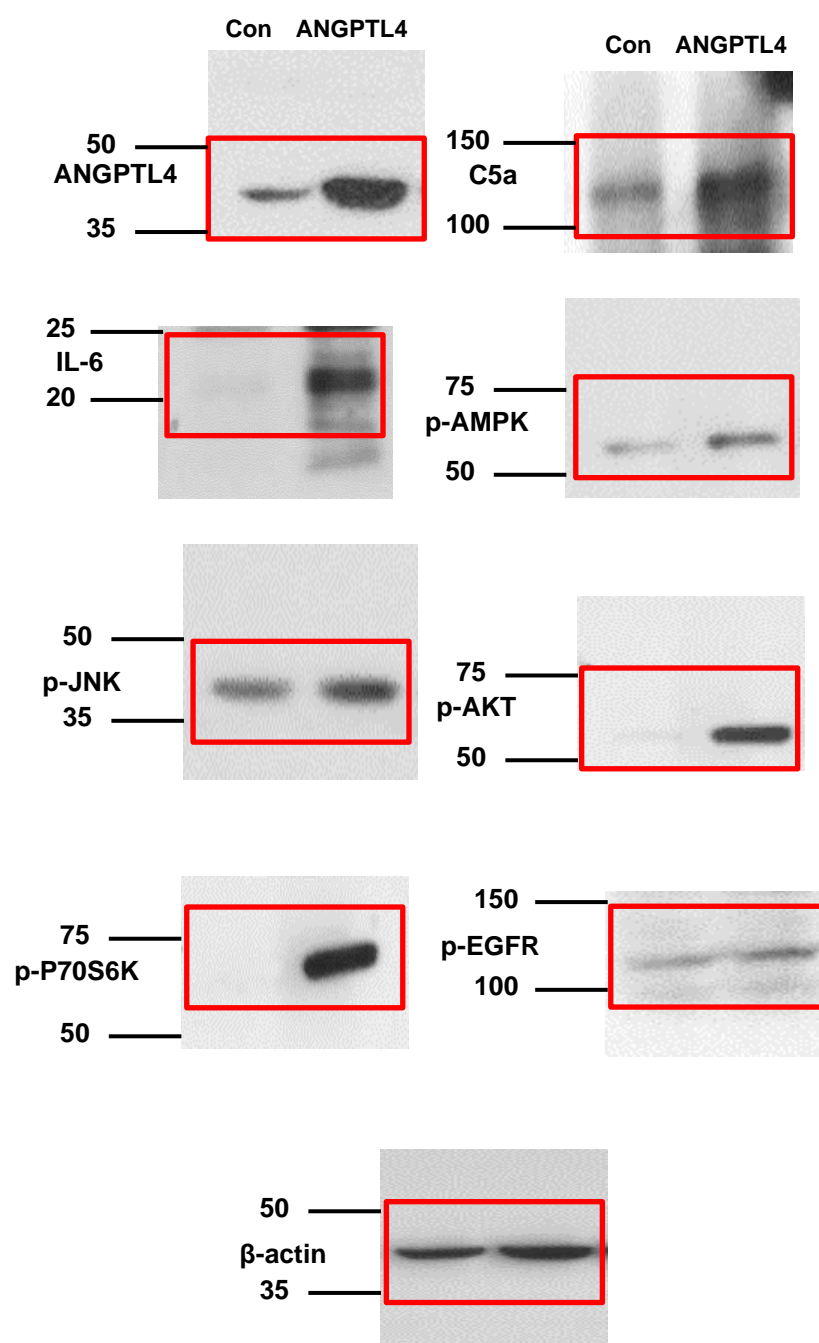

Figure 6D

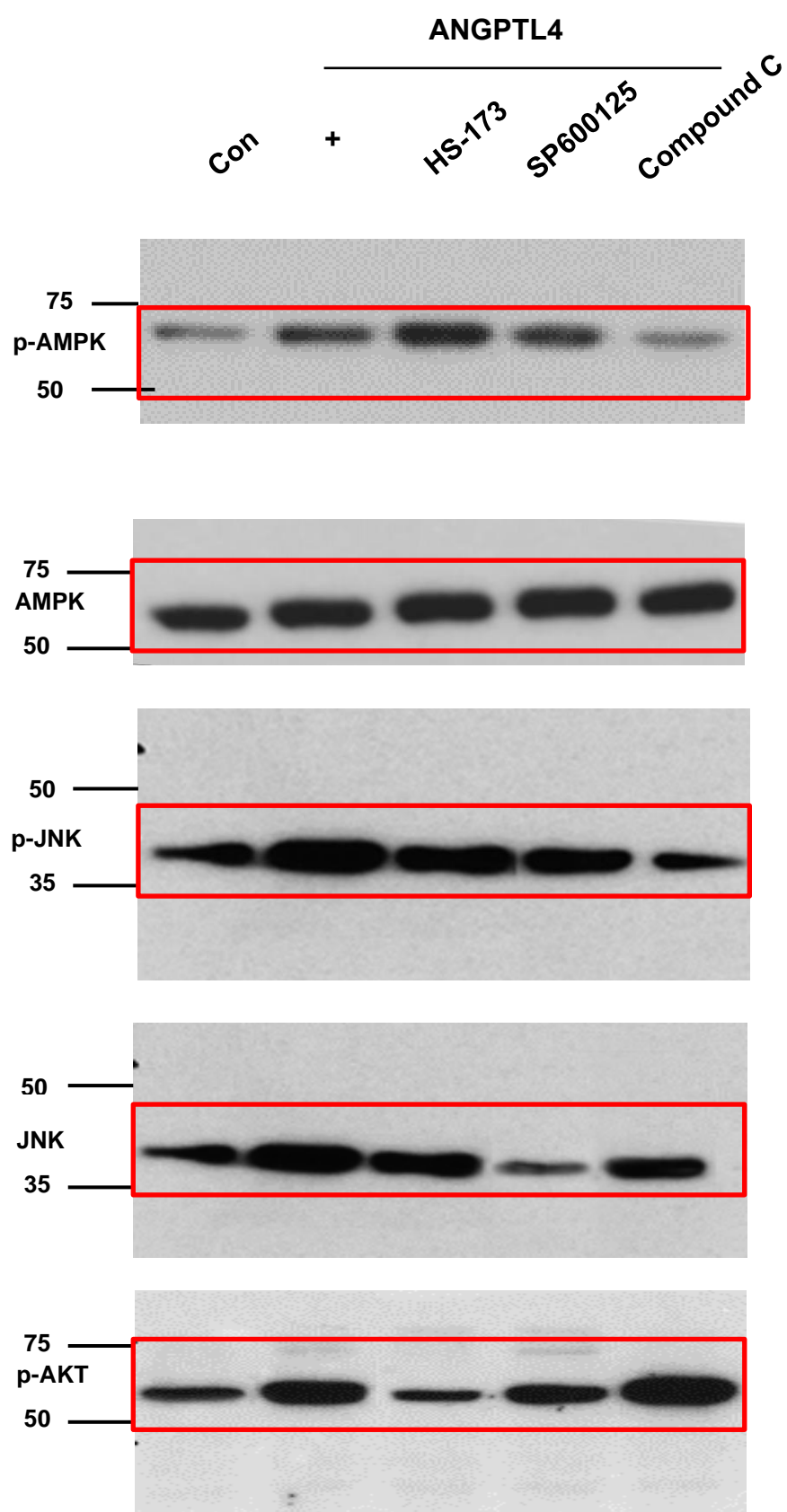

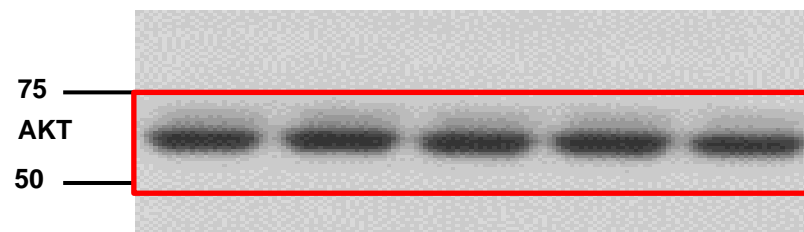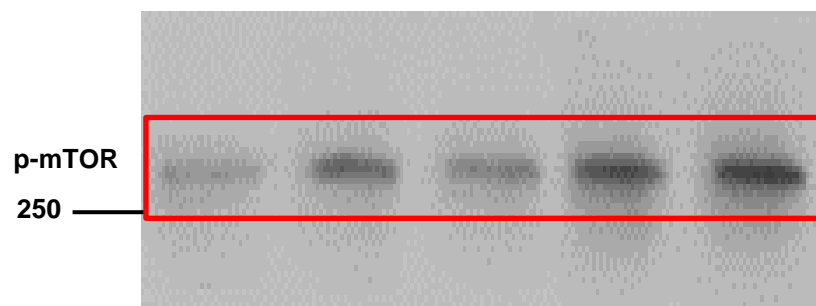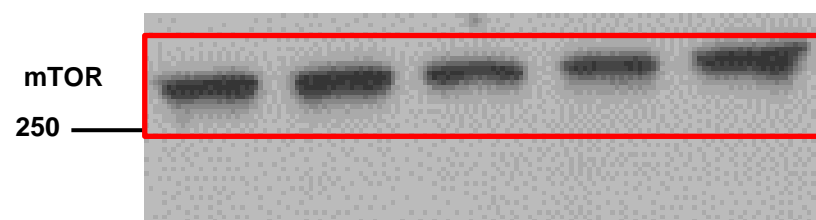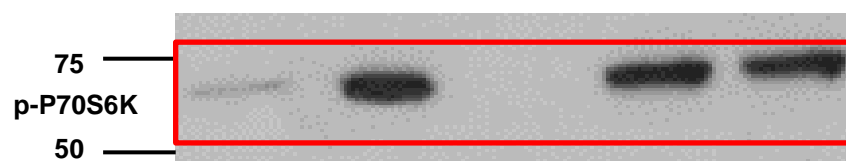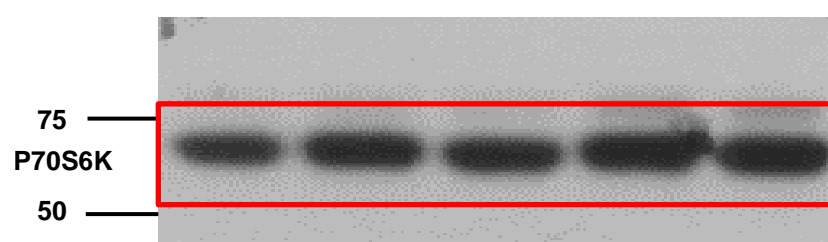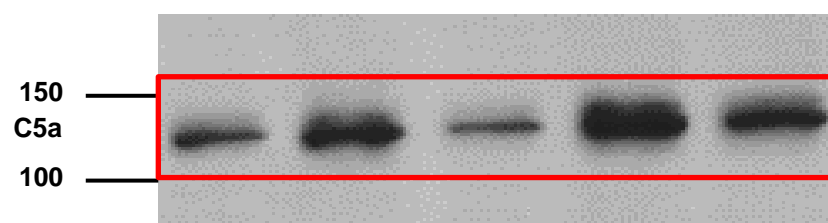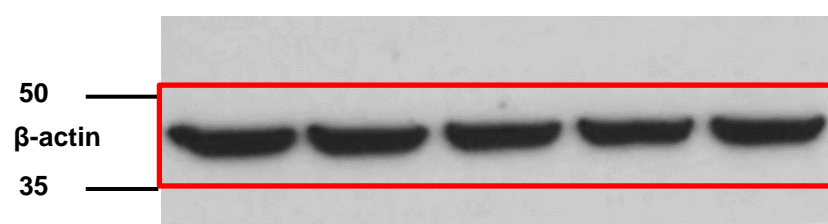

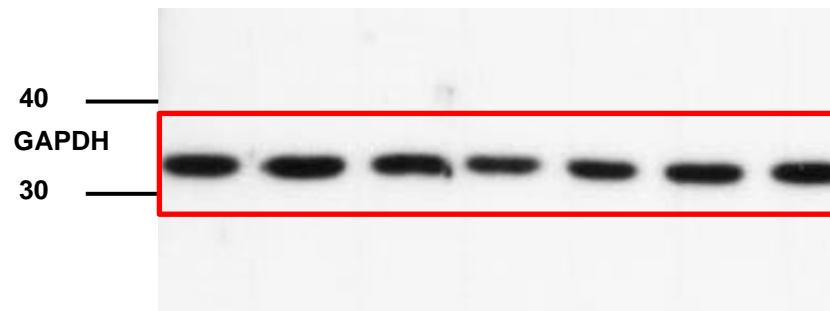

**Figure 6E**

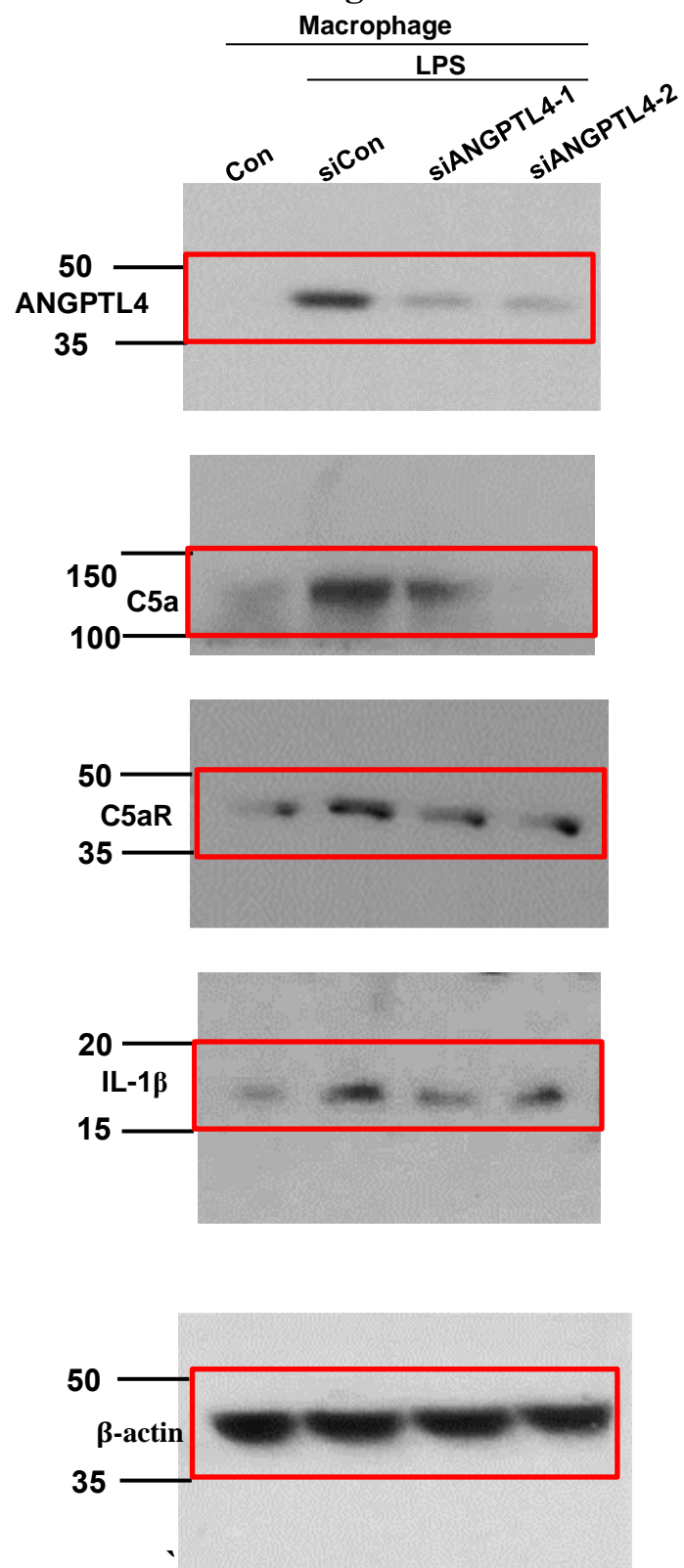

Supplement: Supplementary file 7 — Source Data for Figure 6 [file EMMM-12-e11222-s005.pdf]
